# White matter organization in developmental coordination disorder: A pilot study exploring the added value of constrained spherical deconvolution

**DOI:** 10.1016/j.nicl.2018.101625

**Published:** 2018-12-03

**Authors:** Christian Hyde, Ian Fuelscher, Peter G. Enticott, Derek K. Jones, Shawna Farquharson, Tim J. Silk, Jacqueline Williams, Karen Caeyenberghs

**Affiliations:** a Cognitive Neuroscience Unit, School of Psychology, Deakin University, Geelong, Victoria, Australia; b Cardiff University Brain Research Imaging Centre (CUBRIC), School of Psychology, Neuroscience and Mental Health Research Institute, Cardiff University, UK; c Mary MacKillop Institute for Health Research, Faculty of Health Sciences, Australian Catholic University, Melbourne, Australia; d Melbourne Brain Centre Imaging Unit, Department of Anatomy and Neuroscience, The University of Melbourne, Melbourne, Australia; e Imaging Division, Florey Institute of Neuroscience and Mental Health, Melbourne Brain Centre, Melbourne, Australia; f Developmental Imaging, Clinical Sciences, Murdoch Children's Research Institute, Melbourne, Australia; g Department of Paediatrics, University of Melbourne, Melbourne, Australia; h Institute for Health and Sport, College of Sport and Exercise Science, Victoria University, Melbourne, Australia

**Keywords:** Developmental coordination disorder, DTI, MRI, White matter, Tractography, Motor control

## Abstract

Previous studies of white matter organization in sensorimotor tracts in developmental coordination disorder (DCD) have adopted diffusion tensor imaging (DTI), a method unable to reconcile pathways with ‘crossing fibres’. In response to limitations of the commonly adopted DTI approach, the present study employed a framework that can reconcile the ‘crossing fibre’ problem (i.e., constrained spherical deconvolution- CSD) to characterize white matter tissue organization of sensorimotor tracts in young adults with DCD. Participants were 19 healthy adults aged 18–46: 7 met diagnostic criteria for DCD (4 females) and 12 were controls (3 females). All underwent high angular diffusion MRI. After preprocessing, the left and right corticospinal tracts (CST) and superior longitudinal fasciculi (SLF) were delineated and all tracts were then generated using both CSD and DTI tractography respectively. Based on the CSD model, individuals with DCD demonstrated significantly decreased mean apparent fibre density (AFD) in the left SLF relative to controls (with large effect size, Cohen's *d* = 1.32) and a trend for decreased tract volume of the right SLF (with medium-large effect size, Cohen's *d* = 0.73). No differences in SLF microstructure were found between groups using DTI, nor were differences in CST microstructure observed across groups regardless of hemisphere or diffusion model. Our data are consistent with the view that motor impairment characteristic of DCD may be subserved by white matter abnormalities in sensorimotor tracts, specifically the left and right SLF. Our data further highlight the benefits of higher order diffusion MRI (e.g. CSD) relative to DTI for clarifying earlier inconsistencies in reports speaking to white matter organization in DCD, and its contribution to poor motor skill in DCD.

## Highlights

• All previous diffusion studies of white matter in DCD have employed a tensor model
• We employed a non-tensor model to characterize microstructure in adults with DCD
• The non-tensor model showed atypical white matter organization in the SLF in DCD
• The tensor model failed to detect microstructural group differences for any tract
• Motor impairment characteristic of DCD may be subserved by white matter abnormalities

• We need to move beyond the tensor model in characterizing white matter in DCD

## 1 Introduction

Developmental coordination disorder is a movement disorder that emerges in childhood and persists into adulthood for an estimated 30–70% of those affected (Kirby et al., 2010; Saban and Kirby, 2018). Converging behavioral evidence points towards a possible neural substrate to the reduced motor skill typical of DCD (Biotteau et al., 2016; Gomez and Sirigu, 2015; Wilson et al., 2017). As a result, a series of recent studies have adopted an array of medical imaging techniques to uncover the neural basis of DCD (Biotteau et al., 2016; Fuelscher et al., 2018; Gomez and Sirigu, 2015; Wilson et al., 2017)

Diffusion MRI is one of the most promising techniques to elucidate the functional significance of white matter for motor control, having already done so in healthy controls (Hollund et al., 2018; Johansen-Berg et al., 2007) and in clinical populations, such as stroke (for a review see Puig et al., 2017), cerebral palsy (Glenn et al., 2007; Stashinko et al., 2009; Weinstein et al., 2015), and traumatic brain injury (Caeyenberghs et al., 2011; Caeyenberghs et al., 2010). For example, dexterity and upper limb impairments in stroke patients are often associated with decreased fractional anisotropy (FA) and increased mean diffusivity (MD) in sensorimotor tracts, including the corticospinal tract (CST) and posterior limb of the internal capsule (Puig et al., 2017). Furthermore, recent work suggests that diffusion MRI modelling of white matter tissue provides increased accuracy in the prediction of motor outcomes in patient groups relative to other common imaging methods, such as functional magnetic resonance imaging (*f*MRI) (Qiu et al., 2011; Song et al., 2014).

Until now, only four studies have explored white-matter tract microstructure in DCD (Debrabant et al., 2016; Langevin et al., 2014; Williams et al., 2017; Zwicker et al., 2012). Across these studies, microstructural differences (relative to controls) have been reported in a variety of sensorimotor tracts including the CST, superior longitudinal fasciculus (SLF), inferior longitudinal fasciculus, the internal capsule and corpus callosum (CC). However, there are considerable inconsistencies in those tracts that have been implicated in DCD and results are rarely replicated. We point towards a number of methodological considerations that have likely contributed to these mixed findings.

Firstly, a variety of approaches have been used to analyze diffusion MRI data including manual delineation of 3D tracts via tractography (e.g. Zwicker et al., 2012), automated anatomical labelling atlases (Debrabant et al., 2016), and voxel-based analyses (VBA) methods such as tract-based spatial statistics (TBSS) (Williams et al., 2017). While manual tractography is time consuming and requires anatomical expertise, it typically generates tracts that more closely align with structural anatomy relative to common atlas based and VBA approaches (Van Hecke and Emsell, 2016). For example, during TBSS a tract skeleton/template is placed over the purported center of white matter tracts, where FA is expected to be highest (Kirkovski et al., 2015; Smith et al., 2006). DTI-based diffusion metrics are then assumed to reflect white matter organization at the center portion of the tract/s of interest middle. However, it is now well documented that such templates at times fail to reflect the ‘center’ of a tract which can result in questionable anatomical accuracy, the combination of which can result in spurious group differences in tract metrics (see Bach et al., 2014). Thus, some have argued that TBSS based approaches should be interpreted with caution (Bach et al., 2014; Pecheva et al., 2017; Tamnes et al., 2017).

With respect to behavioral relevance, in some of the above studies alterations in white matter organization coincided with poorer motor performance in the DCD group. However, the direction of the correlation coefficients have also been mixed/unexpected in some studies (Zwicker et al., 2012). For example, Zwicker and colleagues found that the decreased motor ability observed in children with DCD was associated with decreased mean axial diffusivity in the CST and posterior thalamic radiation. However, developmental studies have typically shown that, where changes are observed, axial diffusivity tends to decrease with age from childhood through to adolescence (for a review, see Lebel et al., 2017). This is likely the result of the increased complexity in fibre orientation that is generally observed with maturation, which would hinder diffusion along the principal axis of the diffusion ellipsoid and reduce axial diffusivity. Accordingly, were white matter properties in motor tracts to contribute to poorer motor outcomes, one might assume microstructure to be disrupted which would generally manifest as increased axial diffusivity in otherwise healthy children. Consequently, the behavioral significance of previously reported white matter abnormalities in DCD is difficult to discern.

Finally, in all previous diffusion MRI studies in DCD the diffusion tensor framework has been adopted (i.e., diffusion tensor imaging- DTI), which requires only moderate numbers of encoding directions (a minimum of six) and relatively low *b*-values (*b* ~ 1000 s/mm2) (Caan, 2016). Accordingly, DTI remains the most common diffusion MRI modelling method. Nonetheless, the fundamental limitations of the DTI model have been well documented in seminal works (Farquharson and Tournier, 2016; Farquharson et al., 2013; Jeurissen et al., 2013; Jones, 2008; Jones et al., 2013; Tournier et al., 2011). Briefly, DTI relies on a single tensor to estimate fibre orientation within a voxel, meaning that it can only resolve a single fibre orientation per voxel and is unable to represent multiple fibres in a single voxel. Since fibres are thought to cross, fan and/or diverge in up to 90% of white matter voxels (Jeurissen et al., 2013), it is now widely recognized that the DTI framework is prone to creating anatomically inaccurate reconstructions or spurious white matter tracts/streamlines and should therefore be interpreted with extreme caution (Tournier et al., 2011). Hence, while previous studies applying the basic tensor framework have provided important insight into white matter organization in DCD, these data must be interpreted in the context of the abovementioned fundamental limitations.

In response to limitations of DTI, there have been considerable efforts in recent years to develop higher order non-tensor approaches to diffusion MRI (for a detailed review see Farquharson and Tournier, 2016). One such approach is constrained spherical deconvolution (CSD), a high angular resolution model that is robust to the issue of crossing fibres (Farquharson and Tournier, 2016; Farquharson et al., 2013; Tournier et al., 2008). Accordingly, CSD generates metrics such as apparent fibre density (AFD) (Raffelt et al., 2012a) which is less susceptible to the ‘crossing fibres’ problem (Farquharson and Tournier, 2016; Jones et al., 2013; Raffelt et al., 2012b). CSD-based tractography (Jeurissen et al., 2011) has already been shown to generate anatomically superior reconstructions of white-matter tracts compared with DTI in healthy adults (Auriat et al., 2015; Farquharson et al., 2013) and children (Toselli et al., 2017), as well as in patients with stroke (Auriat et al., 2015), Alzheimer's disease (Reijmer et al., 2012) and neurological motor disorders (Stefanou et al., 2016).

Here we investigated, for the first time, white matter organization in young adults with DCD using a high angular resolution diffusion imaging (HARDI) sequence and CSD. With respect to the latter, in order to control for partial volume effects and reduce isotropic signals (Xu et al., 2017b) we employed the damped Richardson-Lucy (dRL) algorithm (Dell'Acqua et al., 2010). We first aimed to use CSD-based tractography to reconstruct the CST and SLF in young adults with and without DCD and compare white matter metrics across groups. We chose the CST and SLF since multiple studies have reported microstructural abnormalities in DCD in these tracts (e.g., Debrabant et al., 2016; Langevin et al., 2014; Williams et al., 2017), and their respective contribution to motor control is well-established (Carmel and Martin, 2014; Parlatini et al., 2017; Tremblay et al., 2017). Further, confining our focus to these two tracts a priori reduced the risk of false positives associated with ‘whole-brain’ approaches (Van Hecke and Emsell, 2016). Finally, to address the disadvantages of template-based and VBA approaches, we manually delineated these tracts for each participant. We also conducted DTI-based tractography on the same participants in order to compare common diffusion metrics derived across CSD and DTI modelling methods. We hypothesized that individuals with DCD would show atypical white matter organization relative to controls in the CST and SLF (reflected in common diffusion MRI metrics) which would be associated with reduced motor competence.

## 2 Methods

### 2.1 Participants

Consistent with earlier diffusion MRI studies of samples of individuals with DCD, participants were 7 (4 females and 3 Males) who met the DSM-5 criteria for DCD, and 12 controls (3 females and 9 Males) aged between 18 and 46 years. No difference was detected in mean age between the DCD (*M*_Age_ = 23.29; SD_Age_ = 4.31) and control groups (*M*_Age_ = 26.16; SD_Age_ = 7.64). All individuals with DCD and all but one control (who reported being ambidextrous) were self-reported right handers. The ambidextrous control participant, however, showed a right-hand preference during standardized motor assessment (described below). Participants gave written informed consent and the project received ethical approval from the Deakin University Human Research Ethics Committee.

Project advertisements were placed on University websites at an Australian University and on social media (i.e., Facebook). Participants with DCD were screened in accordance with DSM-5 criteria and recent guidelines for identifying DCD in adults (Barnett et al., 2015). Specifically, participants with DCD presented with motor proficiency significantly below the expected age-norm (Criterion A), as indicated by Bruininks-Oseretsky Test of Motor Proficiency (BOT-2: Bruininks, 2005) scores <16th percentile- for a summary of BOT-2 total and sub-scale scores for participants in the DCD group, see Table 1. The BOT-2 is a well-validated standardized measure of motor skill. It was adopted here because recent studies in Australian cohorts and reviews of commonly adopted measures of motor proficiency found it to be the most valid and reliable battery for identifying motor impairment in young adults (Hands et al., 2015; McIntyre et al., 2017). Motor difficulties significantly reduced participants' abilities to undertake daily activities requiring movement (Criterion B) and emerged in early childhood (Criterion C), as determined using the Adult Developmental Co-ordination Disorders/Dyspraxia Checklist (ADC) (Kirby et al., 2010). In the absence of agreement of a reliable cut-off for the ADC, we adopted an approach that we have previously described in detail (Hyde et al., 2014), which has subsequently been used (e.g. Hyde et al., 2018; Kashuk et al., 2017). In an earlier study comprising 47 healthy young Australians (Hyde et al., 2014), we identified the CI_95%_ for the ADC total score (CI_95%_: 21.26 _Mean_ +/− 3.27) and ADC child scale (CI_95%_: 4.26 _Mean_ +/− 0.86). Those participants who met Criterion A (described above) and fell above the CI_95%_ cut off for the Total (i.e., 25 or above) and child (i.e., 6 and above) were deemed to have met criteria B and C respectively. Initially 8 participants had motor skill ≤ the 16th percentile, but one of these failed to meet Criteria B and C as described here. A further participant met Criterion C according to the ADC, but failed to meet Criterion B. However, this participant verbally stated that their poor motor skills negatively impacted their performance of motor related daily tasks so we were confident of their meeting Criterion B and their subsequent inclusion in the DCD group. One participant who met our criteria for DCD had a previous diagnosis of Dyspraxia/DCD. Since all participants were either recruited through the University setting or had completed an undergraduate degree, they were deemed to have had intelligence at least in the normal range (Criterion D). Further, no participants reported a previous diagnosis of a neurological or medical condition affecting movement (e.g., cerebral palsy). Finally, no participant reported a diagnosis of ADHD or similar neurodevelopmental disorder.

**Table 1:** Summary BOT-2 information for participants with DCD

	BOT-2 Total Percentile	BOT-2 Fine Motor Control sub scale	BOT-2 Manual Coordination sub scale	BOT-2 Body Coordination sub scale	BOT-2 Strength and Agility sub scale
DCD	9^th^	10^th^	24^th^	16^th^	12^th^

Controls were free of self-reported medical or neurological impairment. None self-reported motor difficulties and where possible, the motor ability of control participants were assessed using the BOT-2. Accordingly, 8 controls performed the BOT-2 with all presenting with motor ability above the 20th percentile.

### 2.2 MRI acquisition

MR scanning was conducted using a Siemens Skyra 3 T MRI system (Erlangen, Germany) with a 32-channel receive only head coil. High resolution T1-weighted 3D MPRAGE images were acquired for each participant using the following parameters: TR = 1900 ms, TI = 900 ms, TE = 2.49 ms, flip angle = 9°, voxel size = 0.9 mm^3^, acquisition matrix 256 × 256, FoV = 240 mm, 192 contiguous slices with an acquisition time of 4:26 min. High angular resolution diffusion imaging (HARDI) was conducted using a single shot EPI sequence with the following parameters: TR = 8500 ms, TE = 110 ms, flip angle = 90°, voxel size = 2.5 mm^3^, acquisition matrix 96 × 96, FoV = 240 mm, 60 contiguous slices. A total of 64 non-collinear diffusion weighted directions with *b* = 3000 s/mm^2^ were captured, and 8 non-weighted images (*b* = 0 s/mm^2^) interleaved between the weighted images. Acquisition time was 10:32 min. The higher *b*-value adopted here relative to previous studies of white matter organization in DCD was used to improve the resolution of distinct fibre orientations for tractography (Caan, 2016).

### 2.3 Diffusion weighted data pre-processing

Diffusion data were processed using Explore DTI software v4.8.6 (Leemans et al., 2009), embedded within MATLAB R2015b (Mathworks, Natick, MA, USA). First, images were visually inspected using the ExploreDTI quality assurance tools (e.g. looping of diffusion MRI images in differing image planes, inspection of outlier profiles and average residuals for diffusion weighted volumes) to allow for volumes with artefacts to be removed (Coppieters et al., 2018; Ivers et al., 2018; Pijnenburg et al., 2016). Images were then corrected for subject motion and eddy current-induced geometric distortions in native space, with cubic interpolation and the RESTORE approach taken to maximize accuracy (Chang et al., 2005). The latter also incorporated *b*-matrix rotation and echo planar imaging (EPI) correction (Irfanoglu et al., 2012; Ivers et al., 2018; Leemans and Jones, 2009).

### 2.4 Whole-brain tractography

Pre-processed diffusion weighted data were then subjected to whole brain deterministic CSD and DTI tractography in ExploreDTI. In order to control for partial volume effects and reduce isotropic signals (Xu et al., 2017b) we employed the damped Richardson-Lucy (dRL) algorithm (Dell'Acqua et al., 2010). Fibre tractography was generated based on the following parameters: seedpoint resolution was set at 2×2×2 mm, angle threshold at 45°, a fibre orientation distribution (FOD) threshold of 0.05 and fibre length range 10–500 mm (as per Bourbon-Teles et al., 2017). For DTI-based tracking, in order to facilitate comparison of tract metrics across both models, similar angle threshold and fibre length were used for the DTI and CSD frameworks (see Auriat et al., 2015 for a similar approach).

### 2.5 Region of Interest (ROI) analysis

**Fig. 1:**
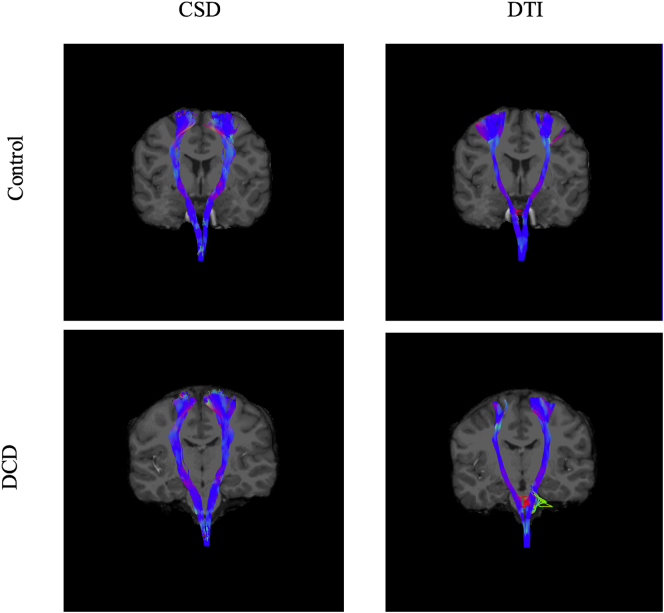
A visual representation of CST tracts using CSD and DTI modelling in a representative individual with DCD and a control participant. NOTE: CST- corticospinal tract; CSD- constrained spherical deconvolution; DTI- diffusion tensor imaging.

**Fig. 2:**
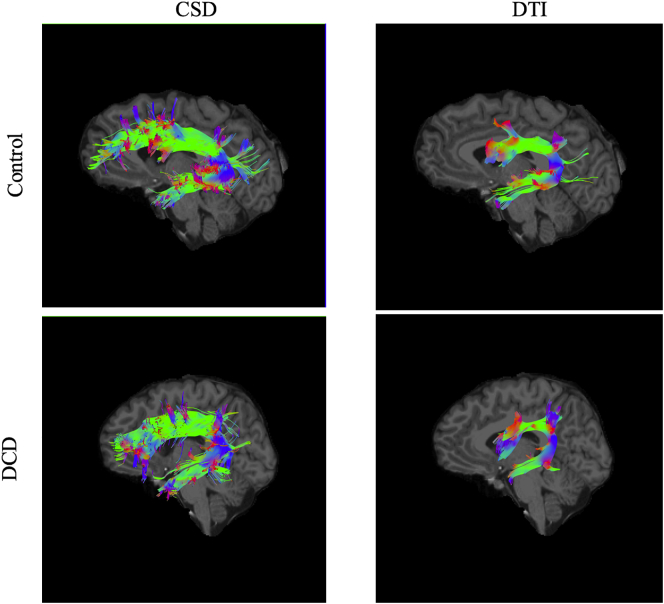
A visual representation of SLF tracts using CSD and DTI modelling in a representative individual with DCD and a control participant. NOTE: SLF –Superior longitudinal fasciculus; CSD- constrained spherical deconvolution; DTI- diffusion tensor imaging.

A visual representation of the fibre projections reconstructed for the CST and SLF can be seen in Fig. 1 and Fig. 2 respectively. ROIs were manually delineated for each individual participant by CH. Specifically, ROIs were drawn in each participant's native space to delineate the CST and SLF bilaterally. Individual participant FA color maps were used to delineate fibre orientations across planes (i.e., coronal, sagittal & axial planes) and ROIs were placed using the highly reproducible methods described in detail by Wakana et al. (2007) (see also Catani and De Schotten, 2008). These methods are commonly used to delineate ROI's for manual tractography (e.g. Cabeen et al., 2018; Coad et al., 2017; Filbey et al., 2014; Paldino et al., 2015).

**Fig. 3:**
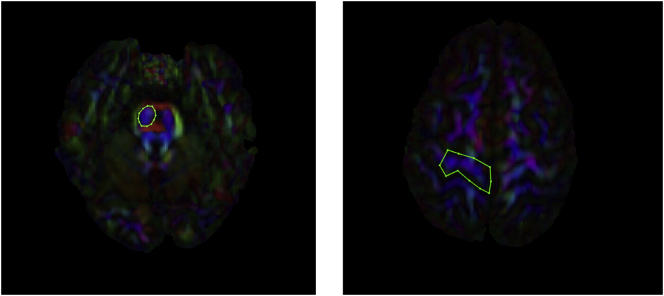
A visual representation of ROI masks for the CST. NOTE: ROI: Region of interest; CST- corticospinal tract.

**Fig. 4:**
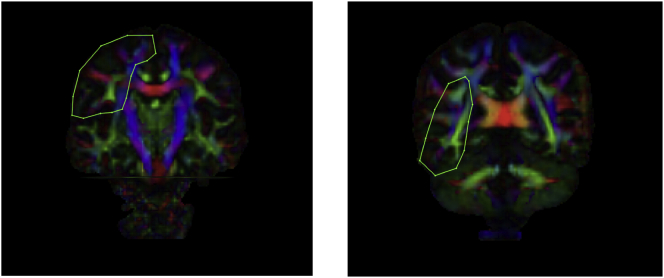
A visual representation of ROI masks for the SLF. NOTE: ROI: Region of interest; SLF- superior longitudinal fasciculus.

The *CST* was reconstructed by placing an ROI in the axial plane at the decussation of the superior cerebellar peduncle followed by a second ROI at the most ventral axial slice where the divide of the central sulcus can be identified (see Fig. 3 for a visual representation of these ROI's). This ROI placement largely reconstructs projections to the medial motor cortices. Lateral projections are difficult to recreate accurately due intersecting association fibres which result in substantial variability in fibre orientation (Wakana et al., 2007). We chose this method because it provides a sound trade-off between the need for specificity and sensitivity (Xu et al., 2017a) and shows excellent inter and intra-rater reliability (Wakana et al., 2007). Accordingly, lateral projections were, for the most part, not generated in the present study. Since there is compelling evidence to suggest that CSD may be better able to reconstruct lateral projections of the CST where DTI is unable or less reliable (Auriat et al., 2015; Farquharson et al., 2013), our choice to adopt ROI's that would more likely recreate the medial branches of the CST also allowed for more direct comparison of diffusion metrics generated for the CST using CSD and DTI respectively. Further, given that the DTI model is less able to reconstruct the lateral portions of the CST, and earlier studies of CST microstructure in DCD adopted the DTI model, our approach also allowed for more direct comparison of CST metrics between the current study and earlier work. The *SLF* was reconstructed by identifying the middle of the posterior limb of the internal capsule in the axial view whereby a coronal slice was selected. An initial ROI was placed on this coronal slice to include the core and all branches of the SLF. Then, the middle of the splenium of the CC was identified in mid-sagittal view whereby a coronal slice was selected and a second ROI placed around all SLF fibres (see Fig. 4 for a visual representation of these ROI's). While there remains some conjecture as to the precise delineation of the SLF in humans, our approach recreated the whole SLF including the arcuate fasciculus in accordance with recent and seminal diffusion works (Kamali et al., 2014; Makris et al., 2004; Wakana et al., 2007) and is common in neuropsychological settings (e.g. Madhavan et al., 2014; Urger et al., 2015).

Exclusion ROIs were applied to remove anatomically spurious fibres when delineating the CST and SLF. As noted, the same inclusion ROI's (as described above) were applied when reconstructing tracts for an individual regardless of whether the CSD or DTI model was used. However, as expected, the two models often produced different fibre projections. Therefore, where anatomically spurious fibres were detected, the incidence and location of spurious fibres often differed across models and hence exclusion ROI's differed. Finally, while all efforts were made to ensure that participant identity was masked while ROI's were delineated, this was not always possible. Though ROI's were applied using the highly reproducible methods described in the methods above (e.g. as per Wakana et al., 2007), we cannot rule out the possibility of unintended or subliminal bias during participant tractography. While this is unlikely to have unduly biased aggregated metrics, we must certainly acknowledge this possibility.

The following diffusion metrics were obtained from the reconstructed fibre tracts: mean tract volume (mm^3^), mean fractional anisotropy (FA), mean diffusivity (MD), mean apparent fibre density (AFD) (Dell'Acqua et al., 2013).

### 2.6 Analysis

In order to compare diffusion metrics between CSD and DTI derived tractography, two-way mixed ANOVAs with Group as the between-subjects factor (2 levels: DCD vs Control) and diffusion MRI model as the within-subject variable (2 levels: CSD vs DTI) were conducted on mean tract volume (mm^3^), mean FA and mean MD for each tract and hemisphere.

To compare those individuals with and without DCD on these metrics, a series of independent samples *t-*tests were conducted on CSD (e.g., mean tract volume, mean FA, mean MD and mean AFD) and DTI derived metrics (e.g., mean tract volume, mean FA, and mean MD) for each tract. We corrected for multiple comparisons using the False Discovery Rate (FDR) of 0.05 (corrected *p* values are denoted as *p*_FDR_) (Benjamini and Hochberg, 1995). Where group comparisons showed significant differences between individuals with and without DCD for a given tract (e.g., mean AFD of the left SLF), Spearman's Rho correlations were conducted against percentile ranking for total BOT-2 scores to determine if there was a relationship between motor performance and diffusion metrics. As noted, the direction of correlations between diffusion metrics and motor ability have been mixed in earlier studies with samples of individuals with DCD (e.g. Zwicker et al., 2012), rendering interpretation difficult. Lower AFD values have been reported in sensorimotor tracts of patients groups where motor impairment is common [e.g., hemiparesis: (Scheck et al., 2015) and adolescents born very pre-term (Raffelt et al., 2013)]. Hence, should diffusion metrics in the SLF be associated with motor ability, we reasoned that lower AFD values would be correlated with lower motor competence. Accordingly, we adopted a one-tailed (i.e., directional) approach for our correlational analysis between mean AFD of the left SLF and percentile ranking for total BOT-2 scores.

Finally, in light of evidence that group differences in diffusion metrics have shown to alter along the length of white matter tracts (e.g. Dodson et al., 2017; Groeschel et al., 2014; Sibilia et al., 2017), it is understood that whole tract average metrics can obscure group differences. Accordingly, along tract analyses were conducted for all tracts reconstructed using CSD and DTI respectively, and group comparisons across points were then conducted. The number of points generated for each tract was calculated by dividing the mean tract length by the voxel size (e.g. 2.5 mm in our case) (see Leemans et al., 2009). Since no systematic group differences were detected, mean tract length was collapsed across groups. The following number of points were calculated for CSD modelled tracts (mean tract length in parentheses): CST left, 47 points (117 mm); CST right, 45 (112 mm); SLF left, 40 (101 mm); SLF right, 38 (94 mm). The following number of points were calculated for DTI modelled tracts (mean tract length is in parentheses): CST left, 47 (118 mm); CST right, 46 (116 mm); SLF left, 33 (83 mm); SLF right, 30 (75 mm). All participants had their SLF and CST fibre projections resampled using these same number of points, and the aforementioned diffusion metrics were generated for each point.

## 3 Results

Two-way ANOVA (group x diffusion MRI model) on mean tract volume, mean FA and mean MD failed to reveal any interaction effects with group for either the left or right CST, or left or right CST. Main effects for diffusion MRI model (CSD vs DTI) are presented in Table 2 for the CST (including descriptive statistics averaged across groups) and Table 3 for the SLF (including descriptive statistics averaged across groups). With respect to the CST, main effects analysis demonstrated significantly lower FA for both the left[*F*(1, 17) = 62.64, *p* < .001, partial η^2^ = 0.79], and right[*F*(1, 17) = 75.61, *p* < .001, partial η^2^ = 0.82] CST as well as increased mean diffusivity in the left CST[*F*(1, 17) = 8.92, *p* = .008, partial η^2^ = 0.34] when parameters were estimated using the CSD model compared to DTI. With respect to the SLF, main effects analysis demonstrated significantly higher tract volume for both the left[*F*(1, 17) = 258.91, *p* < .001, partial η^2^ = 0.94] and right[*F*(1, 17) = 394.37, *p* < .001, partial η^2^ = 0.96] SLF, as well as lower FA for both the left[*F*(1, 17) = 227.94, *p* < .001, partial η^2^ = 0.93] and right[*F*(1, 17) = 126.40, *p* < .001, partial η^2^ = 0.88] SLF when parameters were estimated using the CSD model relative to DTI.

**Table 2:** Mean and SD (in parentheses) diffusion metrics for the CST averaged across group.

		CSD	DTI	*p*
CST _Left_	Mean Tract Volume (mm^3^)	4853 (2023)	5438 (2022)	.452
	Mean FA	519 (.020)	.555 (.017)	<.001
	Mean MD (mm^2^)	5.292^-4^ (.176^-4^)	5.188^-4^ (.162^-4^)	.008
CST _Right_	Mean Tract Volume (mm^3^)	5766 (2277)	5883 (1517)	.869
	Mean FA	506 (.026)	.558 (.016)	<.001
	Mean MD (mm^2^)	5.204^-4^ (.150^-4^)	5.189^-4^ (.203^-4^)	.829

**Table 3:** Mean and SD (in parentheses) diffusion metrics for the SLF averaged across group.

		CSD	DTI	*p*
SLF _Left_	Mean Tract Volume (mm^3^)	33743 (5851)	9841 (3563)	<.001
	Mean FA	.406 (.012)	.469 (.018)	<.001
	Mean MD (mm^2^)	5.095^-4^ (.095^-4^)	5.152^-4^ (.161^-4^)	.134
SLF _Right_	Mean Tract Volume (mm^3^)	36727 (6531)	10102 (4464)	<.001
	Mean FA	.404 (.016)	.465 (.020)	<.001
	Mean MD (mm^2^)	5.012^-4^ (.111^-4^)	5.056^-4^ (.144^-4^)	.166

**Fig. 5:**
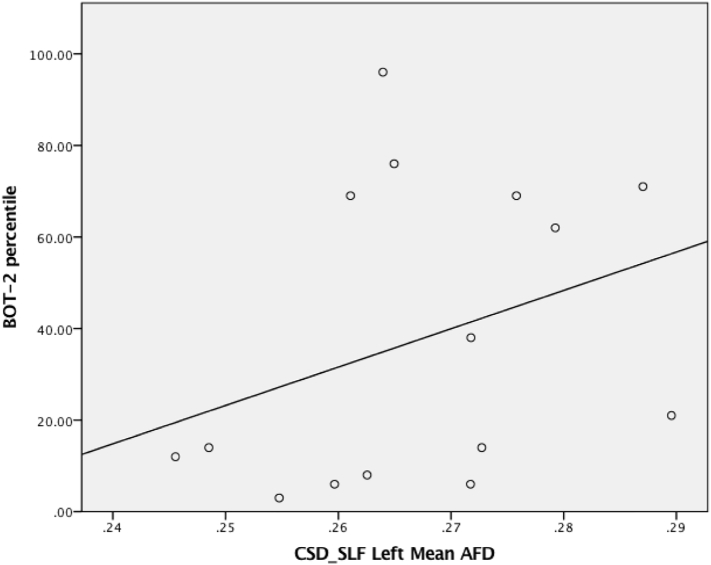
A visual representation of the relationship between mean AFD of the SLF and total BOT-2 percentile ranking. NOTE: AFD- Apparent Fibre Density; SLF –Superior longitudinal fasciculus.

Independent samples *t*-test revealed that mean AFD of the left SLF was significantly lower in individuals with DCD (*M* = 0.259; *SD* = 0.011) than controls (*M* = 0.275; *SD* = 0.013), *t*(17) = −2.70, *p* = .015 (0.039 _FDR_), *d* = 1.32. Further, individuals with DCD (*M* = 33,782; *SD* = 6552) showed a trend for decreased tract volume in the right SLF relative to control (*M* = 38,444; *SD* = 6135) (*p* = .137). No other group comparisons reached statistical significance. For a summary of group comparisons and group descriptive statistics for diffusion metrics, see Table 4 and Table 5 for the CST and SLF respectively. Finally, Spearman's Rho correlation between mean AFD of the left SLF and total BOT-2 percentile score revealed a statistically significant moderate positive correlation, *r*_*S*_ = 0.442, *p* = .049 (See Fig. 5 for a visual representation).

**Table 4:** Descriptive statistics for diffusion metrics for the left and right CST derived from CSD and DTI modelling across groups.

		**CSD**			**DTI**		
		DCD	Control	*p*⁎	DCD	Control	*p*
CST _Left_	Mean Tract Volume (mm^3^)	4718 (1606)	4932 (2296)	.831	5502 (1126)	5401 (2448)	.920
	Mean FA	.522 (.012)	.516 (.518)	.531	.560 (.018)	.552 (.016)	.322
	Mean MD (mm^2^)	5.336 ^-4^ (.151 ^-4^)	5.267^-4^ (.191^-4^)	.421	5.252 ^-4^ (.197 ^-4^)	5.151 ^-4^ (.135 ^-4^)	.200
	Mean AFD	.384 (.017)	.378 (.021)	.521	-	-	-
CST _Right_	Mean Tract Volume (mm^3^)	6094 (2260)	5574 (2364)	.645	6013 (1136)	5808 (1745)	.785
	Mean FA	.516 (.026)	.501 (.025)	.273	.557 (.015)	.559 (.017)	.835
	Mean MD	5.196 ^-4^ (.154 ^-4^)	5.208^-4^ (.155^-4^)	.864	5.194 ^-4^ (.169 ^-4^)	5.186 ^-4^ (.228 ^-4^)	.941
	Mean AFD	.385 (.018)	.375 (.020)	.295	-	-	-

⁎ significant differences between groups at *p* <.05.

**Table 5:** Mean and SD statistics for diffusion metrics for the left and right SLF derived from CSD and DTI modelling across groups.

		CSD			DTI		
		DCD	Control	*p*	DCD	Control	*p*
SLF _Left_	Mean Tract Volume (mm^3^)	34650 (6334)	33213 (5772)	.620	10051 (2954)	9718 (3996)	.851
	Mean FA	.402 (.011)	.408 (.012)	.278	.467 (.013)	.471 (.020)	.686
	Mean MD (mm^2^)	5.103 ^-4^ (.091 ^-4^)	5.090^-4^ (.101^-4^)	.797	5.135 ^-4^ (.125 ^-4^)	5.161 ^-4^ (.183 ^-4^)	.743
	**Mean AFD**	**.259 (.011)**	**.275 (.013)**	**.015**⁎**(.039**_**FDR**_**)**	-	-	
SLF _Right_	**Mean Tract Volume (mm**^**3**^**)**	**33782 (6552)**	**38444 (6135)**	**.137**	9227 (4493)	10613 (4564)	.529
	Mean FA	.408 (.019)	.402 (.014)	.409	.463 (.015)	.466 (.023)	.719
	Mean MD	5.021 ^-4^ (.106 ^-4^)	5.007^-4^ (.119^-4^)	.809	5.029 ^-4^ (.130 ^-4^)	5.071 ^-4^ (.144 ^-4^)	.548
	Mean AFD	.269 (.021)	.269 (.012)	.972	-	-	

⁎ significant differences between groups at *p* <.05.

**Fig. 6:**
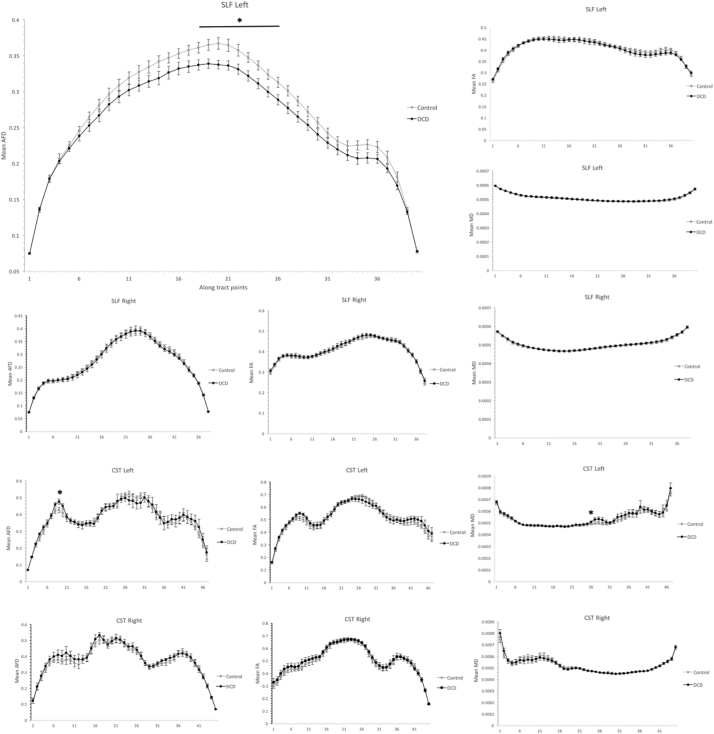
Along tract values for CSD modelled SLF and CST tracts, including mean AFD, FA and MD. Error bars reflect standard error. * Represents points where group differences reached statistical significance at *p* <.05 uncorrected. No comparisons survived FDR correction. NOTE: CSD- constrained spherical deconvolution; SLF, superior longitudinal fasciculus; CST, corticospinal tract; AFD, apparent fibre density; FA, fractional anisotropy; MD, Mean Diffusivity.

**Fig. 7:**
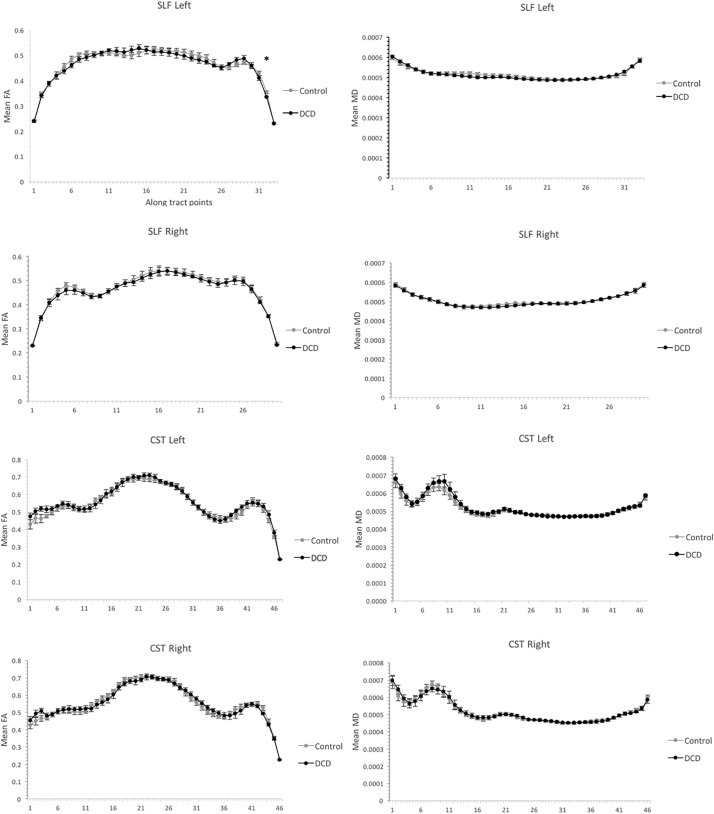
Along tract values for DTI modelled SLF and CST tracts, including mean FA and MD. Error bars reflect standard error. * Represents points where group differences reached statistical significance at *p* <.05 uncorrected. No comparisons survived FDR correction. NOTE: DTI- Diffusion tensor imaging; SLF, superior longitudinal fasciculus; CST, corticospinal tract; FA, fractional anisotropy; MD, Mean Diffusivity.

Along tract group comparisons for each tract modelled using CSD and DTI are summarised in Fig. 6 and Fig. 7 respectively. For CSD modelled tracts, other than mean AFD in the left SLF, no consistent group differences were observed. That is, in all cases except the former, either no or one group comparison (of a possible 33–47 depending on the tract) were found to be statistically significant at *p* < .05 uncorrected, and no group comparisons survived FDR correction. Thus, while no group differences were observed for whole tract mean values for the CST bilaterally and the right SLF on mean AFD, FA or MD, these effects were consistent across the length of the tract. Conversely, for the left SLF, those with DCD showed significantly lower mean AFD in 9 consecutive tract points (18–26 inclusive) relative to controls. While none of these comparisons survived FDR correction, the data show a clear trend for lower mean AFD of the SLF that was select to, or accentuated, immediately anterior to the arcuate fasciculus. Finally, no systematic along tract group differences were observed for any DTI modelled tract.

## 4 Discussion

This study was the first to investigate white matter organization in young adults with DCD using CSD-based tractography. As predicted, young adults with DCD showed divergent white matter organization to controls in the SLF, with CSD modelling revealing significantly decreased AFD in the left SLF relative to controls (with large effect size, *d* = 1.32), and a trend towards reduced tract volume in the right SLF (with medium-large effect size, *d* = 0.73). Interestingly, DTI-based reconstruction of SLF tracts failed to reveal any group differences and no group differences were observed for the CST regardless of tract laterality or diffusion MRI model (i.e., CSD or DTI). Given the methodological advantages afforded by CSD relative to DTI (e.g. addressing the ‘crossing fibre’ issue) and that all previous accounts of white matter organization in DCD have adopted DTI, our study highlights the added value of CSD as a method for clarifying previously inconsistent findings speaking to the profile of white matter organization in DCD. Taken together, these data are broadly consistent with the view that the motor difficulties symptomatic of DCD may be subserved by microstructural tissue differences in sensorimotor tracts, specifically the SLF. They further highlight the benefits of moving beyond the tensor framework for characterizing white matter tissue properties in DCD. These conclusions and their implications are discussed next.

### 4.1 White matter organization in young adults with DCD

As per earlier work using DTI in children (Langevin et al., 2014) and young adults (Williams et al., 2017) with DCD, CSD-based tractography revealed that white matter organization in the SLF differs between young adults with DCD and controls. Indeed, we observed that mean AFD of the left SLF was significantly reduced in those with DCD, with along tract analysis suggesting that this group difference was accentuated in those portions of the SLF immediately anterior to the arcuate fasciculus. AFD provides a metric of intra-axonal volume fraction (Raffelt et al., 2012a, Raffelt et al., 2012b), with higher values potentially reflecting increased axon diameter or local axon count (Genc et al., 2018). Accordingly, our data suggest that individuals with DCD may have decreased axon diameter or count in the left SLF relative to controls. This is interesting since smaller axon diameter has been associated with slower axonal conduction (Horowitz et al., 2015). We also observed a non-significant trend with a medium-large effect size suggesting reduced tract volume in the right SLF in individuals with DCD. Subsequent analysis suggested that white matter tissue organization in the SLF may be associated with motor ability in our sample. Specifically, we observed that decreased AFD within the left SLF was associated with lower percentile ranking on the total BOT-2 scores. Our findings are consistent with neuroanatomical accounts attesting to the broader role of the SLF in motor control (Parlatini et al., 2017; Tremblay et al., 2017). Furthermore, the SLF provides connections between dorsal regions that support higher order sensorimotor processing, as well as non-motor functions including executive processing and language (Forkel et al., 2014; Parlatini et al., 2017). Accordingly, our data here supports a recent ALE meta-analysis demonstrating that cortical activation in several of those sensorimotor regions serviced by the SLF (e.g., the middle frontal gyrus, superior frontal gyrus supramarginal gyrus and inferior parietal lobule) is reduced in those with DCD during tasks of manual dexterity (Fuelscher et al., 2018). Still, given the broader role of the SLF in non-motor functions and that the symptom profile of those with DCD is complex and often includes symptoms beyond the motor domain, it is also possible that differences in white matter organization in the SLF between those with and without poor motor function might have implications beyond the motor domain including contributing to common executive control issues.

It is interesting to note that group differences in mean AFD were select to the left hemisphere. We speculate that one reason for this effect may be because motor control is lateralized to the hemisphere contralateral to the executing limb and that all participants were right handed. Further, fronto-parietal systems (which are serviced by the SLF) in the left hemisphere appear to be particularly important for the representation of purposive actions (often referred to as praxis), regardless of handedness (Króliczak et al., 2016). Given that action representation and praxis are both purported to be impaired in DCD (Reynolds et al., 2015), the finding of disorganization within the left SLF is perhaps unsurprising.

While previous evidence is mixed, those studies that have reported differences in SLF microstructure between individuals with and without DCD did so using DTI-based tractography. While we detected group differences in SLF white matter organization in adults with DCD using the CSD-based tractography, unlike earlier work we failed to do using DTI-based tractography. Below we elaborate on methodological considerations which we argue likely account for differences in the CSD based analyses of the present study to DTI based analyses of earlier work.

No significant differences in diffusion metrics were observed in the CST between those with and without DCD regardless of diffusion model (DTI vs CSD). This finding sits in the context of mixed findings in earlier work. While one previous study included young adults with DCD (Williams et al., 2017), the remainder involved children (Debrabant et al., 2016; Zwicker et al., 2012). Thus, we must be circumspect when comparing neuroanatomical findings of children and adults (for all tracts) regardless of whether they share a common diagnosis. Still, Williams et al. (2017) did report differences in diffusion metrics for the CST between young adults with and without DCD which contrasts to our study. Using DTI, Williams and (2017) adopted a TBSS approach to compare diffusion metrics between groups. As detailed earlier, when TBSS is applied diffusion metrics are drawn from a skeleton/template which is thought to reflect the center of white matter tracts (Kirkovski et al., 2015; Smith et al., 2006). However, data should be interpreted with caution given that such templates often do not reflect the ‘center’ of a tract, and offer limited anatomical accuracy (for a detailed discussion see Bach et al., 2014). Taken with the broader limitations of the DTI framework (used by all previous studies in DCD), we argue that our manually delineated CSD-based modelling of the CST provides one of the more robust accounts of white matter organization in the CST in young adults with DCD.

Given the well-established role of the CST in supporting motor performance, our finding of preserved white matter organization in young adults with DCD may seem counter-intuitive at first. However, even in severe developmental neurological disorders of movement such as cerebral palsy (CP), reports of CST damage are common at the group level (Gordon, 2016), yet inconsistent at the individual participant level. For example, in a sample of 28 individuals with CP, Stashinko et al. (2009) reported that <30% presented with CST abnormalities relative to >95% showing abnormalities within the posterior thalamic radiation. Thus, even in childhood motor disorders with a more clearly defined neurological basis than DCD, it is not uncommon for the CST to either be preserved or for abnormalities to vary considerably in extent. With this in mind, the motor difficulties characteristic of CP are typically more severe than those observed in DCD. Indeed, some have suggested that the two disorders may exist on a continuum of motor impairment with DCD at the less extreme end (Pearsall-Jones et al., 2010; Williams et al., 2014). It is therefore reasonable to expect that the underlying neuropathology of motor impairment in DCD may be more subtle and/or inconsistent than in CP. This may in part explain our inability to detect microstructural differences within the CST in our sample of young adults with DCD.

### 4.2 Comparison of CSD and DTI representations of white matter microstructure in DCD: the advantages of higher order approaches and directions for future work

The findings reported here parallel evidence suggesting that CSD produces anatomically superior tracts, and may be more sensitive for detecting white matter abnormalities in patient groups (e.g. Auriat et al., 2015; Reijmer et al., 2012). Indeed, as per earlier accounts (e.g. Auriat et al., 2015), CSD modelling detected group differences in microstructure (here for the SLF) that DTI was unable to detect. Further, our analyses demonstrated that CSD modelling produced higher tract volume than DTI for the SLF, and significantly lower FA values in all tracts than DTI modelling (Auriat et al., 2015; Reijmer et al., 2012; Stefanou et al., 2016). CSD derived tracts often show increased tract volume relative to DTI, in part due to the ability of CSD to track fibres through voxels with crossing fibres which results in larger tracts that more closely align with anatomy (Reijmer et al., 2012; Stefanou et al., 2016). These effects are most pronounced in those tracts with a high proportion of crossing fibres (e.g. the SLF). Similarly, FA values tend to be lower for CSD derived tracts, reflecting the decreased uniformity of diffusion in voxels with crossing fibres (Auriat et al., 2015), the latter of which cannot be reconciled by the tensor model. With increased anatomical accuracy comes more powerful and sensitive designs. In support, the differences in SLF volume observed here between those with and without DCD based on CSD modelling were not replicated by DTI-based tractography. Further, we observed group differences in AFD of the SLF, a metric that cannot be derived from the tensor model, providing additional evidence of the potential for increased sensitivity offered from non-tensor approaches. Finally, by reducing the incidence of generating spurious reconstructions and providing a more anatomically representative recreation of white matter tracts, CSD reduces the risk of false positives relative to DTI (i.e. incorrectly reporting differential white matter connectivity in a sample of individuals with DCD) (Farquharson and Tournier, 2016).

We argue that the aforementioned limitations of DTI have likely contributed to previously inconsistent finding speaking to the role of white matter connectivity in reduced motor skill in DCD. Further, while our sample size is in-keeping with available diffusion studies using samples of individuals with DCD, we acknowledge that it is nonetheless modest. Regardless of this, however, since ours is the first study to model white matter organization in DCD using CSD we argue that our data provide one of the more robust accounts of white matter organization in DCD, at least as it pertains to young adults. Most importantly perhaps, our direct comparison of CSD and DTI derived diffusion metrics, highlights the added value of higher order models in characterizing white matter properties in DCD and the need to move beyond the tensor.

The current sample involved adults with DCD. As eluded to above, the developing brain is not simply a ‘mini’ adult brain, and thus we must be circumspect about generalizing the findings of the present study to children and adolescents with DCD. Indeed, the trajectory of white matter maturation is often varied depending on the tract, with commissural (e.g. CC) and projection (e.g. CST) tracts reaching maturation by early adolescence (e.g. 10 to 15 years), while association fibres (e.g. SLF) follow a more protracted developmental period spanning into early adulthood (Geeraert et al., 2018). Taken with the interplay between sex, genetics and experiential factors that then influences white matter maturation at the individual level (Geeraert et al.), it may certainly be the case that the nature of microstructural differences between children with and without DCD alters as a function of age and sex. This highlights the need for continued investigation into the time course of white matter maturation in children with DCD using higher order diffusion approaches.

While theoretically justified, we opted for a conservative approach in identifying two tracts of interest given the exploratory nature of the study. Hence, while our data are consistent with the view that poor motor skill in DCD may be subserved by white matter tissue organization, they can only speak to potential role of the SLF and CST. Should future larger-scale studies occur, a more detailed account of alternative tracts (e.g. corpus callosum, thalamic radiation) may shed light on the broader role of microstructural tissue properties in DCD symptomatology. The degree to which these contribute to the psychosocial corollaries of impaired motor function should also be the subject of future work.

Future studies should consider a fixel based analysis (FBA) framework to distinguish macroscopic morphology of fibre bundles, including the density of axons within a particular fibre and the change in the cross-sectional area perpendicular to the corresponding fibre bundle (Raffelt et al., 2017). However, given our (theoretically driven) a priori decision to confine tract reconstruction to the SLF and CST, and given that FBA is generally implemented as a data driven whole brain analysis framework, we opted against FBA in the present study. Indeed, while we were adequately powered to detect the hypothesized group differences using tract-based ROI analysis, it is likely that we would have been underpowered to detect group differences at the whole brain level in light of our modest sample size.

Finally, while this study has highlighted some of the benefits of higher order diffusion sequences over tensor models, we should acknowledge that the former are not without limitation. Specifically, a critique of spherical deconvolution methods has been the over-representation of potentially spurious fibre orientations (Dell'Acqua et al., 2010) and the use of a single-fibre response function, which may not be constant across the brain (Dell'Acqua and Tournier, 2018). Indeed, with respect to the latter, evidence suggests that differences in the axonal diameter distributions of white matter tracts may affect the diffusion signal across the brain (Aboitiz et al., 1992), thus leading to inaccurate estimates. However, it is for the most part accepted that assuming a fixed response function represents a relatively benign approximation, particularly at moderate to high *b*-values such as those used in the present study (Dell'Acqua and Tournier, 2018). Similarly, with respect to the estimation of potentially spurious fibre orientations, the present study employed the dampened Richardson-Lucy algorithm, which has been shown to successfully reduce isotropic partial volume effects, and thus guard against potentially spurious fibre orientations (Xu et al., 2017b). Accordingly, while it is important to be mindful of the possible shortcomings of CSD analysis, it is reasonable to expect that these limitations would not have unduly influenced the results of the present study.

## 5 Conclusion

In sum, this study was the first to adopt a higher order approach to white matter tractography in young adults with DCD. Our finding of reduced AFD in the left SLF and tract volume in the right SLF supports the view that abnormal white matter organization may subserve DCD symptomatology, at least with respect to the SLF. In support, lower mean AFD in the left SLF was associated with reduced motor skill as measured via the BOT-2. Further, our comparison of CSD and DTI derived metrics is in-keeping with earlier suggestion that CSD offers increased anatomical accuracy relative to the more commonly adopted DTI model. As such, we argue that our data are well placed to clarify earlier inconsistencies in reports of the contribution of white matter organization in sensorimotor tracts to poor motor skill in DCD, at least with respect to the SLF. More specifically, our work paves the way for continued higher order diffusion modelling of white matter organization in DCD as a means of comprehensively characterizing microstructure in this group, and highlights the need to move beyond the DTI framework.

## Funding sources

KC is supported by a Career Development Fellowship from the National Health and Medical Research Council; PGE is funded by a Future Fellowship from the Australian Research Council; DKJ is supported by a Wellcome Trust Investigator Award (096646/Z/11/Z) and a Wellcome Trust Strategic Award (104943/Z/14/Z).
